# Structural basis of dimerization and nucleic acid binding of human DBHS proteins NONO and PSPC1

**DOI:** 10.1093/nar/gkab1216

**Published:** 2021-12-14

**Authors:** Gavin J Knott, Yee Seng Chong, Daniel M Passon, Xue-hai Liang, Evelyne Deplazes, Maria R Conte, Andrew C Marshall, Mihwa Lee, Archa H Fox, Charles S Bond

**Affiliations:** School of Molecular Sciences, The University of Western Australia, Crawley, WA 6009, Australia; School of Molecular Sciences, The University of Western Australia, Crawley, WA 6009, Australia; School of Molecular Sciences, The University of Western Australia, Crawley, WA 6009, Australia; Department of Core Antisense Research, IONIS Pharmaceuticals Inc., 2855 Gazelle Court, Carlsbad, CA 92010, USA; School of Chemistry and Molecular Biosciences, The University of Queensland, St Lucia, Qld 4072, Australia; Randall Centre for Cell and Molecular Biophysics, King’s College London, New Hunt’s House, Guy’s Campus, London SE1 1UL, UK; School of Molecular Sciences, The University of Western Australia, Crawley, WA 6009, Australia; Department of Biochemistry and Genetics, La Trobe Institute for Molecular Science, La Trobe University, Bundoora, Vic 3086, Australia; School of Molecular Sciences, The University of Western Australia, Crawley, WA 6009, Australia; School of Human Sciences, The University of Western Australia, Crawley, WA 6009, Australia; School of Molecular Sciences, The University of Western Australia, Crawley, WA 6009, Australia

## Abstract

The *Drosophila* behaviour/human splicing (DBHS) proteins are a family of RNA/DNA binding cofactors liable for a range of cellular processes. DBHS proteins include the non-POU domain-containing octamer-binding protein (NONO) and paraspeckle protein component 1 (PSPC1), proteins capable of forming combinatorial dimers. Here, we describe the crystal structures of the human NONO and PSPC1 homodimers, representing uncharacterized DBHS dimerization states. The structures reveal a set of conserved contacts and structural plasticity within the dimerization interface that provide a rationale for dimer selectivity between DBHS paralogues. In addition, solution X-ray scattering and accompanying biochemical experiments describe a mechanism of cooperative RNA recognition by the NONO homodimer. Nucleic acid binding is reliant on RRM1, and appears to be affected by the orientation of RRM1, influenced by a newly identified ‘β-clasp’ structure. Our structures shed light on the molecular determinants for DBHS homo- and heterodimerization and provide a basis for understanding how DBHS proteins cooperatively recognize a broad spectrum of RNA targets.

## Graphical Abstract

**Graphical Abstract:**
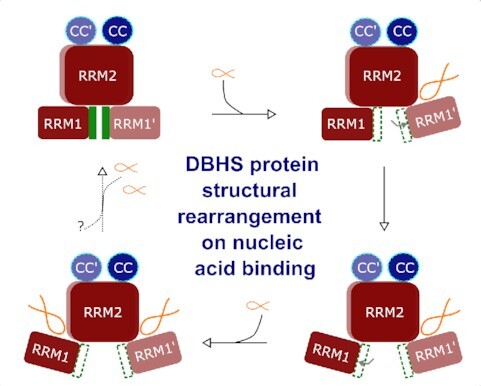
Binding of nucleic acids by DBHS protein dimers involves RRM1 and RRM2 of each monomer. Although the two identical binding sites in a homodimer are not linked, independent rearrangement of RRM1 domains to accommodate nucleic acid requires the unfastening of the N-terminal β-clasp.

## INTRODUCTION

The *Drosophila* behaviour/human splicing (DBHS) proteins are multifunctional gene regulatory RNA/DNA binding proteins (1,2) implicated in tumorigenesis (3–5), innate cellular immune responses (6–9) and neurological development (10–15). In humans, there are three conserved paralogues: non-POU domain-containing octamer-binding protein (NONO, also known as P54^nrb^), paraspeckle protein component 1 (PSPC1) and splicing factor proline/glutamine rich (SFPQ, also known as PSF). Members of the DBHS protein family are defined by a conserved domain architecture, the ‘DBHS region’, consisting of tandem N-terminal RNA recognition motifs (RRM1 and RRM2), a NonA/paraspeckle (NOPS) domain and a C-terminal coiled coil (CC). DBHS proteins exist as obligate dimers, each protomer interacting reciprocally with its partner to create a network of hydrophobic and electrostatic contacts between the NOPS domain, RRM2′ (prime denotes partner chain) and the distal CC′ (14,16–19). More recently, biophysical experiments demonstrated that DBHS protein dimerization is dynamic, with protomers able to form combinatorial hetero- or homodimers at cellular concentrations (14).

DBHS RRMs are reported to bind a diversity of single-stranded RNA (20–23), the conserved 5′ splice site (23, 24) and structured nucleic acids such as the U5 snRNA stem-loop (25). SFPQ interacts directly with double-stranded DNA to exert its role in transcriptional regulation in complex with NONO (26–28). The DBHS proteins also interact with the paraspeckle long non-coding RNA (lncRNA) *NEAT1* (29,30), the lncRNA *MALAT1* (31), the non-coding RNA lncUSM-*MycN* (3) and viral RNA (7,8). Studies of potentially therapeutic modified nucleic acids [e.g. phosphorothioate-containing antisense oligonucleotides (PS-ASOs)] have revealed that they interact with DBHS proteins, causing relocalization from paraspeckle bodies upon transfection (32,33) and hepatotoxicity that may be related to DBHS protein mislocalization and depletion due to tight binding of certain PS-ASOs in a sequence- and chemistry-dependent manner (34). However, the variety of PS-ASO binding proteins suggests a variety of potential binding modes. The only crystal structure to date of such a complex is a PS-ASO bound to the DNA binding domain (DBD) of transcription factor PC4 with a PS-ASO (35) (PDB code: 6YCS), which has a completely unrelated structure to the DBHS proteins. New BRET-based tools for *in vivo* measurement of affinities of proteins for ASOs have revealed the potential for both RRM domains of NONO to be required for ASO binding (36); however, the integral role of RRM2 in dimerization of DBHS proteins indicates the value of a more detailed structural analysis. Indeed, nuclear magnetic resonance investigations indicate that RRM1, and not RRM2, can bind a 5′ splice site RNA fragment (37).

Targeting DBHS proteins for therapeutic benefit in cancers such as neuroblastoma (3) or developmental disorders (12) is limited by a lack of insight into their dimerization states and mechanisms of nucleic acid binding. Here, we describe the crystal structures of the human NONO and PSPC1 homodimers, revealing the structural plasticity of dimerization to provide a rationale for dimer selectivity between DBHS paralogues. In addition, solution X-ray scattering and accompanying biochemical experiments describe a mechanism of cooperative RNA recognition by the NONO homodimer. Our structures describe the molecular determinants for DBHS homo- and heterodimerization and provide a basis for understanding how DBHS proteins cooperatively recognize a broad spectrum of nucleic acid targets.

## MATERIALS AND METHODS

### Expression and purification of the NONO homodimer

Recombinant NONO homodimer was purified, crystallized and the structure solved as described elsewhere (38). Briefly, NONO aa53–312 (NONO-DBHS), aa146–312 (NONOΔRRM1), aa67–312 (NONOΔβ-clasp), aa53–147 (RRM1), aa67–147 (RRM1Δβ-clasp) and the NONO mutant (D183S, R184S, R186S) (NONOΔβ2–β3) (all relative to UniProt entry NONO_HUMAN) were cloned into the first expression cassette of a pET-Duet1 vector (Novagen) and expressed in Rosetta2 (DE3) *Escherichia coli* (Novagen). NONO was purified using column-based nickel affinity chromatography with an N-terminal tobacco etch virus (TEV) protease-cleavable hexahistidine tag. Tagged NONO was subjected to TEV digestion, reverse immobilized metal affinity chromatography and developed over a gel filtration column into 20 mM Tris–Cl (pH 7.5), 250 mM KCl, 50 mM l-proline and 0.5 mM EDTA.

### Expression and purification of the PSPC1 homodimer

Recombinant PSPC1 (aa61–320, relative to UniProt entry PSPC1_HUMAN) was cloned into the first expression cassette of a pET-Duet1 vector (Novagen) (BamHI and EcoRI underlined in sequence) (5′-CAG GAT CCA **GAA AAC CTG TAT TTT CAG GGC** ATG GGG TTC ACT ATC GAC ATC-3′) (5′-CGG AAT TCT TAC ATT AGC ATT AAT TGG TG-3′) with an inserted TEV protease site (bold). Competent *E. coli* Rosetta2 (DE3) cells (Novagen) were transformed and selected from lysogeny broth (LB) agar plates (100 μg ml^–1^ ampicillin, 50 μg ml^–1^ chloramphenicol). Single colonies were inoculated into 5 ml LB (100 μg ml^–1^ ampicillin, 50 μg ml^–1^ chloramphenicol) and incubated for 16 h at 310 K/180 rpm. The 5 ml culture was used to inoculate 500 ml LB (50 μg ml^–1^ ampicillin, 50 μg ml^–1^ chloramphenicol) in 2-l conical flasks incubated at 310 K/180 rpm. At an optical density (600 nm) of 0.6, expression was induced with 0.5 mM IPTG for 16 h at 298 K/180 rpm. Compact pellets of 500 ml were gently resuspended on ice with 50 ml buffer [50 mM Tris–Cl (pH 7.5), 300 mM NaCl, 5 mM imidazole, 10% (v/v) glycerol]. Lysis was carried out with an Emulsiflex C5 high-pressure homogenizer (Avestin) clarified by centrifugation (24 000 × *g*/30 min/278 K) and 0.22 μm filtration before application to a 5 ml NiCl_2_ charged Hi-Trap column (GE Healthcare). PSPC1 elutes over a 10-column volume imidazole gradient [50 mM Tris–Cl (pH 7.5), 300 mM NaCl, 1 M imidazole, 10% (v/v) glycerol]. Peak fractions were subjected to buffer exchange into gel filtration buffer [50 mM Tris–Cl (pH 7.5), 300 mM NaCl, 10% (v/v) glycerol] using a PD-10 desalting column (GE Healthcare) and then incubated at 277 K for 16 h with in-house produced recombinant TEV protease. Post-digestion re-application to a Hi-Trap column (GE Healthcare) removed His-tagged species before concentrated samples were loaded to a HiLoad 16/60 Superdex 200 column (GE Healthcare) developed with gel filtration buffer at 1 ml min^–1^. Purified PSPC1 homodimer concentrated in a 10-kDa concentrator (Amicon) was flash frozen with liquid nitrogen for long-term storage at 193 K.

### Crystallization and X-ray data collection of the NONO and PSPC1 homodimer

Details of the crystallization, data collection and processing for NONO are described elsewhere (38). PSPC1 crystals were obtained by 24-well hanging-drop vapour diffusion experiments in 2:1 ratios of protein at 6.0 mg ml^–1^ to 100 mM (d/l) malic acid (pH 7.0), 28% (v/v) PEG 3350 and 100 mM Tris (pH 7.0) equilibrated against 500 μl reservoir. PSPC1 crystals were flash frozen in liquid nitrogen without cryoprotection and diffraction experiments carried out at the MX2 beamline of the Australian Synchrotron (Melbourne, Victoria, Australia) (39,40). PSPC1 data were processed in XDS (41) with the data merged and scaled using AIMLESS (42). Data collection statistics for NONO and PSPC1 are summarized in Table 1.

**Table 1.** Data collection and refinement statistics

	PSPC1 homodimer	NONO homodimer
**Data collection**		
Space group	*P*12_1_1	*P*12_1_1
Cell dimensions		
*a*, *b*, *c* (Å)	61.54, 63.49, 67.80	67.15, 407.18, 68.96
*α*, *β*, *γ* (°)	90.00, 98.06, 90.00	90.00, 97.75, 90.00
Resolution (Å)	19.41–3.17 (3.54–3.17)^a^	48.15–2.60 (2.65–2.60)^a^
*R*_merge_	0.200 (0.590)	0.088 (0.742)
*I*/*σ_I_*	5.7 (1.65)	10.1 (1.60)
CC (1/2)	0.994 (0.508)	0.995 (0.524)
Completeness (%)	97.7 (98.0)	98.9 (97.7)
Redundancy	3.6 (3.7)	3.7 (3.8)
Wilson *B*-factor (Å^2^)	56.0	48.7
**Refinement**		
Resolution (Å)	19.41–3.17 (3.54–3.17)	48.15–2.60 (2.65–2.60)
No. of reflections	8738 (2390)	110 365 (5520)
*R*_work_/*R*_free_	23.7/28.9	19.7/23.4
No. of atoms		
Protein	4112 (2 monomers)	25 063 (12 monomers)
Ligand/ion		60
Water		194
Average *B*-factor (Å^2^)	59.0	65.0
RMS deviations		
Bond lengths (Å)	0.01	0.01
Bond angles (°)	1.13	1.17

^a^Values in parentheses denote data for the highest-resolution shell.

### Crystallographic structure solution, refinement and validation

The crystal structure of NONO (aa53–312) was phased by molecular replacement as described elsewhere (38). The crystal structure of PSPC1 (aa61–320) was solved by molecular replacement with PHASER (43) with the PSPC1 chain of the PSPC1/NONO heterodimer [PDB code: 3SDE (16)]. Molecular replacement with an ensemble of PSPC1 domains (ensemble 1: residues 66–153; ensemble 2: residues 154–320) found two monomers within the asymmetric unit, consistent with solvent content analysis (44). The resulting model was subjected to iterative model building with COOT (45) and refinement with BUSTER (46). The structures of both the NONO and PSPC1 homodimers were validated using MOLPROBITY (47) and submitted to the Protein Data Bank under the accession codes 5IFM and 5IFN, respectively. Final refinement statistics are included in Table 1.

### Oligonucleotide sample preparation

Fluorescently labelled (5′-6-FAM) homo-ribopolymers (5′-_d_TGGGGGGGGG-3′, 5′-_d_TAAAAAAAAA-3′, 5′-_d_TUUUUUUUUU-3′ and 5′-_d_TCCCCCCCCC-3′) were synthesized by Integrated DNA Technologies (IDT). Fluorescently labelled ASOs were provided by IONIS Pharmaceuticals Inc. (USA) and synthesized by IDT (USA). ASOs IONIS742093 and IONIS626823 are entirely phosphorothioate-modified 5-10-5 ‘gapmers’ modified at the 2′-ribose position with an α-fluoro or constrained ethyl (cET), respectively. Oligonucleotides were prepared in nuclease-free water (Sigma) at 2 mM and diluted with the appropriate RNA binding buffer prior to use.

### Microscale thermophoresis

Microscale thermophoresis (MST) experiments were carried out using a Monolith NT.115 (NanoTemper Technologies) at ambient temperature. Fluorescent oligonucleotides were prepared with *C*_A_ = 100 nM (5′-_d_TGGGGGGGGG-3′ at 500 nM) and incubated for 15 min on ice with a dilution series of NONO titrated in RNA binding buffer [20 mM Tris–Cl (pH 7.5), 250 mM KCl, 50 mM l-proline, 0.5 mM EDTA, 0.05% (v/v) Tween 20, 1 mg ml^–1^ heparin]. The reactions were transferred directly to standard-treated capillaries (NanoTemper Technologies) before primary capillary scans to confirm constant fluorescence between capillaries. Thermophoresis experiments were carried out at 20–25% blue LED (5′-_d_TGGGGGGGGG-3′ at 50% blue LED power) and 20% MST power. Data were modelled using the ‘Thermophoresis + T-Jump’ profile fit to the Hill equation [*f*(*c*) = unbound + (bound – unbound)/(1 + (EC_50_/*c*)*^n^*), with the EC_50_ and *n*_H_ calculated in the NT Analysis software (NanoTemper Technologies).

### Small-angle X-ray scattering data collection and processing

Small-angle X-ray scattering (SAXS) data were collected using size-exclusion chromatography-coupled synchrotron small-angle X-ray scattering (SEC-SY-SAXS) (48) controlled by a Shimadzu HPLC system on the SAXS/WAXS beamline of the Australian Synchrotron with continuous data collection on a 1 M Pilatus detector (49) at 289 K. IONIS742093 at 1 mM was injected in 200 μl and developed at 0.5 ml min^–1^ over a WTC-030N5 (Wyatt) column in 20 mM Tris–Cl (pH 7.5), 250 mM KCl, 50 mM l-proline and 0.5 mM EDTA. NONO protein alone (aa53–312) at 7.0 mg ml^–1^ developed as above was previously published (38). For the NONO:ASO complex, NONO (aa53–312) at 7.0 mg ml^–1^ (115 μM dimer) was complexed with a 1:2.4 molar excess of IONIS742093 for 30 min on ice before SEC-SY-SAXS as described earlier. The data collection parameters and calculated structural parameters are presented in Supplementary Table S1. The scattering data from the first 20 frames were averaged and used for background correction in SCATTERBRAIN. Data were further corrected for cumulative capillary fouling using the US-SOMO HPLC SAXS module (50) before primary data processing using the ATSAS software package (51). The Guinier region, intensity at 0 [*I*(0)] and radius of gyration (*R*_g_) were calculated using PRIMUS (52) with frames averaged where these values were constant. The *P*(*r*) distribution plot, Porod volume and maximum dimension (*D*_max_) were calculated using GNOM (53). The normalized Kratky, Kratky–Debye and Porod–Debye plots were generated in SCÅTTER (BIOISIS) using the flexibility analysis. Theoretical scattering curves and amplitudes were derived from structures and their agreement with scattering data calculated using CRYSOL (54). The structural neighbours of observed solution scattering data were retrieved using the DARA web server (55). The molecular weights of NONO (aa53–312) and IONIS742093 were calculated from the mass parameter *Q*_R_ in SCÅTTER using the volume of correlation (*V*_c_) (56). SAXS data and fits were deposited at SASBDB (57) under the accession codes SASDMR6, SASDMS6, SASDMT6 and project 1489.

### *Ab initio* and rigid body SAXS modelling

For modelling, a high-*q* cut-off of 0.2 was applied due to the decreasing signal-to-noise ratio. *Ab initio* models were generated by 60 DAMMIF (58) modelling runs with *P*2 symmetry imposed. Resulting models were superposed and averaged with DAMAVER (59) before final refinement in DAMMIN (58). Rigid body modelling of the NONO:ASO complex was carried out using SASREF (59) with *P*2 symmetry imposed. Coordinates were manipulated with PDB-MODE (60). The protein component of the starting model was derived from chains AB of the NONO homodimer crystal structure with all heteroatoms removed. A conformer of the bacterial group II intron NMR structure (PDB code: 2M57) was used as a rigid body to describe IONIS742093. Two distance restraints were applied: (i) a range of 5.0–7.5 Å (A144–S147) was used to describe the flexibility of the linker between RRM1 and RRM2 in each monomer; and (ii) a range of 3.5–10.0 Å between F111 of RRM1 and any part of 2M57 was used to describe the requirement of RRM1 for binding. Molecular graphics were created in PYMOL (Schrödinger, LLC).

## RESULTS

### DBHS dimer interfaces reveal structural plasticity that drives dimer selectivity

**Figure 1.**
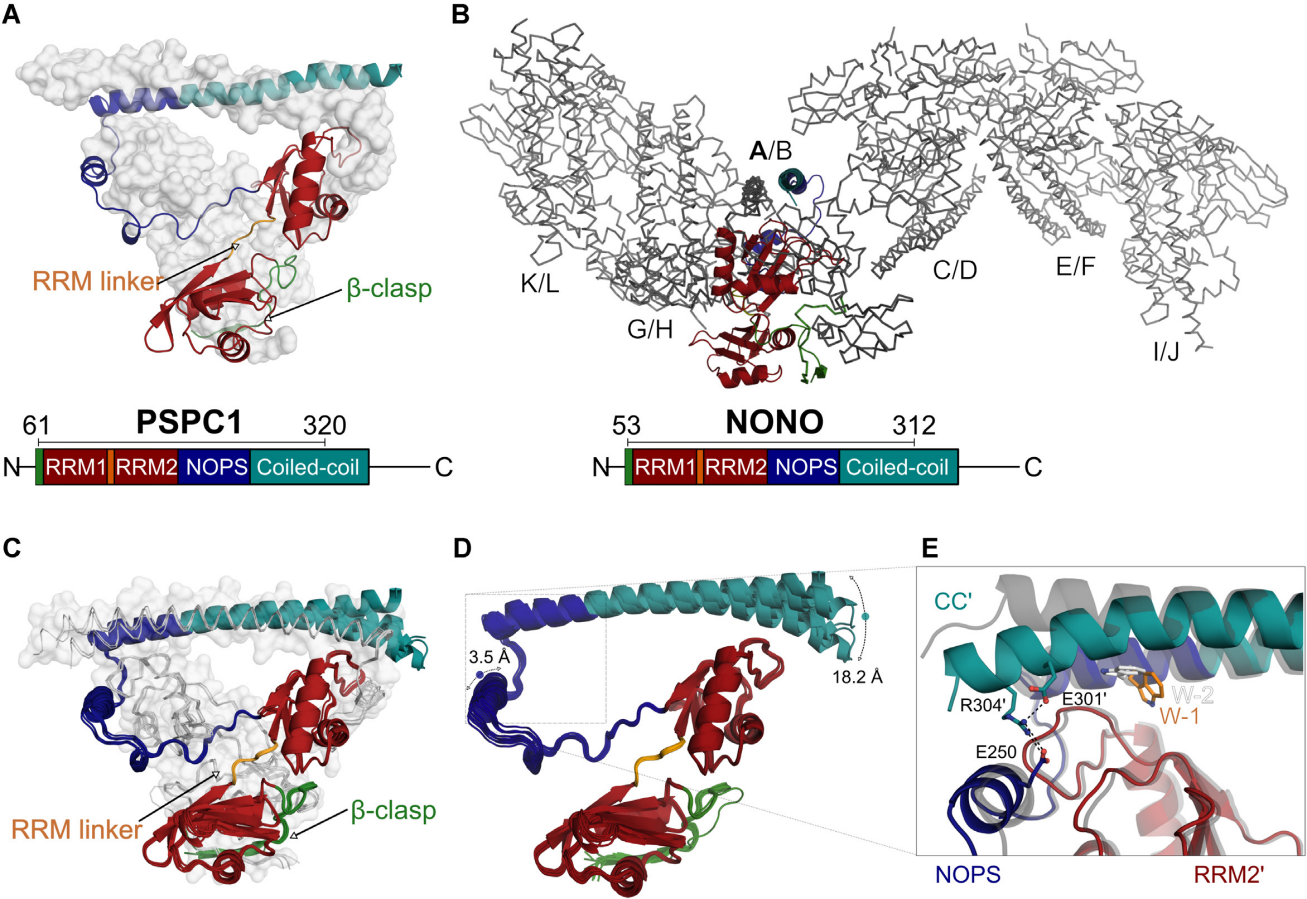
Overall structure of the human PSPC1 and NONO homodimers. (**A**, **B**) The crystal structure of PSPC1-DBHS (61–320) and NONO-DBHS (53–312) homodimers, respectively. The domains crystallized are indicated schematically: β-clasp (green), RRM1 (red), RRM linker (orange), RRM2 (red), NOPS (blue) and CC (teal). (**C**) The six NONO dimers (chains AB, CD, EF, GH, IJ and KL) within the asymmetric unit superposed with an average RMSD of 0.46 Å between dimers (514/520 Cα-atoms). (**D**) The 12 NONO chains within the asymmetric unit overlaid. The variable positioning of the distal CC (teal) and the NOPS domain (blue) is indicated with ranges of motion. (**E**) The two conformers of W271, W_1_ (coloured) and W_2_ (transparent grey), overlaid and viewed from the dimerization interface between the NOPS domain (blue) and partnered RRM2′ (red) and CC (teal). All figures are coloured consistently throughout the manuscript.

To better understand how DBHS proteins function and provide a platform for therapeutic targeting, we determined structures of the conserved DBHS regions of previously uncharacterized human PSPC1 (aa61–320) and NONO (aa53–312) homodimers (PSPC1-DBHS and NONO-DBHS). Both paralogues crystallized as obligate homodimers with two protomers forming highly symmetrical dimers (Figure 1A and B; Table 1). The asymmetric unit of the PSPC1 homodimer is comprised of one dimer; in contrast, NONO is best described as a superhelical array of six highly symmetrical dimers sharing the same domain arrangement (Figure 1B and C).

Given the extensive non-crystallographic symmetry within the asymmetric unit of the NONO-DBHS crystal structure, we wondered whether the related copies might provide insight into the conformational landscape accessible to the NONO homodimer. Pairwise comparisons of monomers or dimers revealed conformational differences that were isolated to the NOPS and the distal region of the CC domain (Figure 1D and E). To explore the correlation between movements in the NOPS and distal CC, we grouped monomers of NONO by RMSD within the NOPS domain (Supplementary Table S2). Two clusters were observed and made distinct by the conformation of W271, a highly conserved residue positioned within the hydrophobic dimer interface necessary for DBHS protein dimerization and paraspeckle localization (16). The first cluster, designated W_1_, is described by W271 adopting a conformation where the plane of the indole engages in a pocket formed within RRM2′ (Figure 1E, orange). In contrast, the second cluster, designated W_2_, is described by the indole of W271 stacking above RRM2′ (Figure 1E, white). Examining the conformational variation in the distal CC′ revealed that it is correlated with the conformation of W271 and that of the NOPS domain (Figure 1E). Moreover, the asymmetric unit of the NONO-DBHS crystal structure features W271 conformationally symmetric dimers (W_1_/W_1_ and W_2_/W_2_) and conformationally asymmetric dimers (W_1_/W_2_) (Supplementary Table S2).

**Figure 2.**
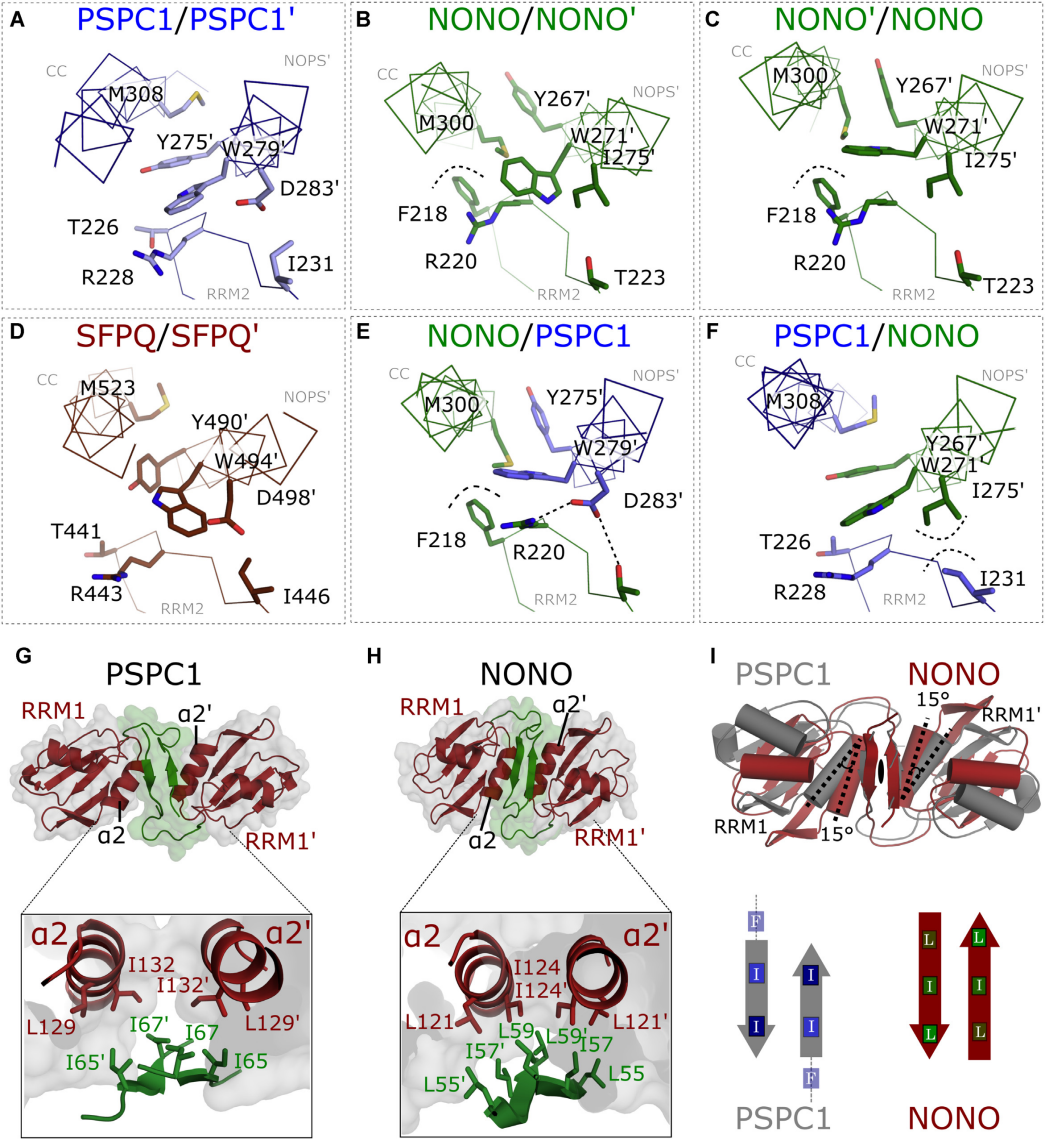
Conformational plasticity of conserved residues at DBHS protein dimer interfaces, including a ‘β-clasp’ structure. (**A–F**) The core dimerization interface of crystallized DBHS dimers shown in a ribbon representation projected along the CC domain. Conserved residues are drawn in a stick representation. (**G**, **H**) Side-by-side comparison of the NONO and PSPC1 β-clasp. (**I**) Superposition of the NONO and PSPC1 RRM1 domains relative to the 2-fold rotation axis centred at the β-clasp.

Given the observed sampling of alternative conformations by NONO within the dimerization interface, we wondered whether energetically favourable remodelling of the dimerization interface determines preferred DBHS dimerization states. Six distinct DBHS dimeric combinations are possible; however, heterologous expression and purification support an intrinsic preference for heterodimerization (14,18,61,62). To explore this further, we compared the interfaces of homodimeric PSPC1 and NONO with the published PSPC1/NONO (16) and homodimeric SFPQ (18). While architecturally consistent (Supplementary Figure S1), the NOPS′:CC:RRM2 interface shows distinct residue-dependent conformational variability between dimerization states (Figure 2A–F). The interface formed by DBHS homodimers is dominated by CC interactions between conserved Trp, Tyr and Met packed above RRM2 (Figure 2A–D). The heterodimeric interface of PSPC1/NONO is asymmetrical in conformation, where NONO contributing a bulky hydrophobic, F218, is coupled to favourable electrostatics between R220, T223 (RRM2, NONO) and D283′ (NOPS, PSPC1) (Figure 2E). In contrast, the opposing interface features an additional hydrophobic collapse between I275′ (NOPS′, NONO) and I231 (RRM2, PSPC1) (Figure 2F). Taken together, upon PSPC1/NONO heterodimerization the NOPS′:CC:RRM2 interface is remodelled to accommodate varied residues to produce a net sum of favourable interactions. Given position matched residues in SFPQ, a heterodimer formed of SFPQ/NONO would present a similarly favourable set of interactions at the NOPS′:CC:RRM2 interface. Taken together, the PSPC1 and NONO homodimers provide a wealth of insight into the structural plasticity and general flexibility of the dimerization interface, providing a structural basis for favourable heterodimerization.

The PSPC1 and NONO homodimers possess an additional antiparallel two-stranded β-sheet formed by the N-terminal strand of each subunit that clasps both RRM1 and RRM1′ in a 2-fold symmetrical arrangement (Figure 2G–I). The β-sheet, hereafter referred to as the β-clasp, generates a tight hydrophobic core packed against a pair of amphipathic helices at the base of RRM1, α2 and α2′. Superposing the β-clasps present in the PSPC1 and NONO homodimers reveals a shifted register and symmetrical twist by 15° of the PSPC1 RRM1 domain (Figure 2I, grey) relative to NONO (Figure 2I, coloured).

### DBHS proteins bind nucleic acid with an absolute requirement for RRM1

**Figure 3.**
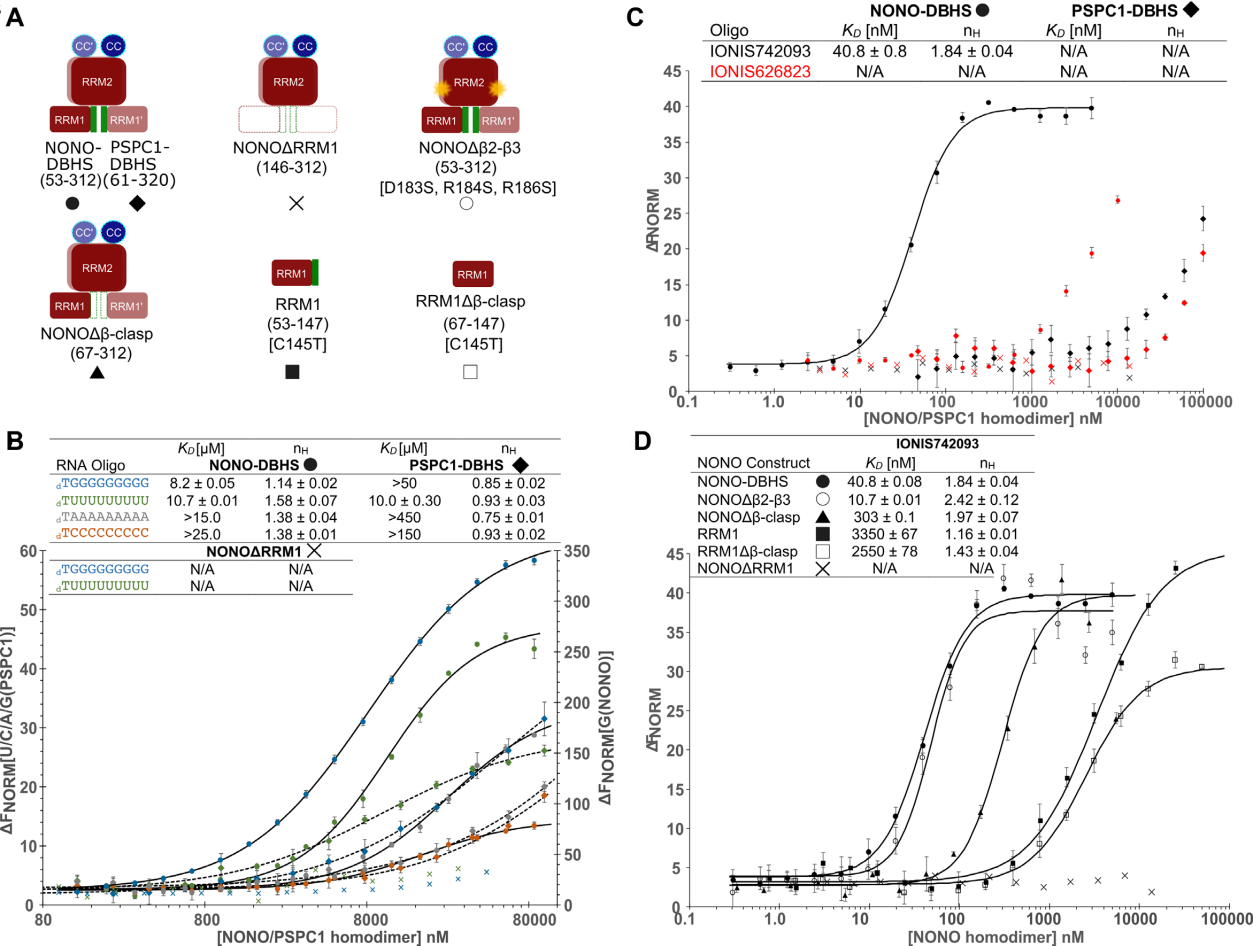
RRM1 is essential for nucleic binding and NONO and PSPC1 homodimers have different nucleic acid specificities. (**A**) Schematic representation of NONO/PSPC1 constructs used in this study. (**B**) MST binding curves for NONO/PSPC1 interacting with single-stranded homo-ribonucleic acids. Baseline-corrected normalized fluorescence (Δ*F*_NORM_) for polyG (blue) interacting with NONO is plotted on the right and Δ*F*_NORM_ for polyU/C/A (green/grey/orange) and polyG interacting with PSPC1 on the left against concentration of NONO/PSPC1 in nM. The binding coefficient *K*_D_ (μM) and Hill coefficient (*n*_H_) are summarized in a table. (**C**) Binding curves for NONO/PSPC1 interacting with 2′-modified ASOs. Δ*F*_NORM_ is plotted against concentration of NONO/PSPC1 in nM. The binding coefficient *K*_D_ (μM) and Hill coefficient (*n*_H_) are summarized in a table. (**D**) Binding curves for constructs of NONO interacting with IONIS742093, where Δ*F*_NORM_ is plotted against concentration of NONO in nM. The binding coefficient *K*_D_ (μM) and Hill coefficient (*n*_H_) are summarized in a table.

A hallmark of DBHS protein function is the broad recognition of single-stranded RNA sequences in a variety of cellular processes (1). To assess DBHS protein RNA binding *in vitro*, we performed MST using recombinantly expressed and purified DBHS protein constructs (Figure 3A) to measure binding to fluorescently labelled oligonucleotides (Figure 3B). NONO homodimer shows a preference for G- or U-rich homo-ribopolymers, binding with low micromolar affinity (*K*_D_ = 8.2 and 10.7 μM, Figure 3B). In contrast, NONO lacked affinity for C- or A-rich homo-ribopolymers with affinity constants >25 μM. PSPC1-DBHS, in comparison, demonstrated binding to only U-rich homo-ribopolymers (*K*_D_ = 10 μM, Figure 3B). Interestingly, these data are best described using the Hill equation, where the Hill coefficient (*n*_H_) ≥1 suggests cooperativity. To test the importance of RRM1 in nucleic acid binding, we truncated NONO-DBHS to remove RRM1 (NONOΔRRM1) (Figure 3A). Despite forming a stable dimer containing RRM2 (Supplementary Figure S3), NONOΔRRM1 no longer demonstrated any association with the homo-ribopolymer targets (Figure 3B).

To explore the role of RRM1 in binding to high-affinity RNA targets, we tested the reported *in vivo* association between 2′-modified PS-ASOs and NONO (32,33,36). NONO-DBHS bound with low nanomolar affinity to a 2′-α-fluoro-PS-ASO (2′-F-PS-ASO; IONIS742093) (*K*_D_ = 40.8 nM, *n*_H_ = 1.84) and showed high micromolar affinity for a 2′-cET-PS-ASO (IONIS626823) (Figure 3C), consistent with *in vivo* observations. In contrast, PSPC1-DBHS did not exhibit any significant affinity for either IONIS742093 or IONIOS626823 (Figure 3C). As with binding to homo-ribopolymers, NONO-DBHS binding to IONIS742093 was undetectable in the absence of RRM1 (Figure 3D).

We next wondered whether our structure could inform us on the molecular basis for DBHS protein RNA recognition. Removing the N-terminal β-clasp (NONOΔβ-clasp) and assaying binding to IONIS742093 reduced the *K*_D_ to 303 nM without affecting the Hill coefficient (*n*_H_ = 1.97) (Figure 3D). Thus, the β-clasp appears to be required for nucleic acid binding, but not cooperativity in binding. Within RRM2, the β2–β3 loops have been suggested as RNA binding due to their remarkably high sequence conservation (17); however, mutation of NONO-DBHS to produce NONOΔβ2–β3 had no effect on binding to IONIS742093 (Figure 3D). To test whether RRM1 could function as an RNA binding domain outside of the context of a dimer, we purified RRM1 and RRM1Δβ-clasp in isolation and observed that neither were sufficient for high-affinity or cooperative binding to IONIS742093 (Figure 3D). Taken together, these data suggest that more than one molecule of nucleic acid is able to associate with the DBHS region cooperatively. Furthermore, RRM1 is necessary but not sufficient for RNA binding, suggesting that NONO must form a functional dimer for high-affinity RNA binding.

### Solution scattering of NONO, IONIS742093 and NONO:IONIS742093 complex

**Figure 4.**
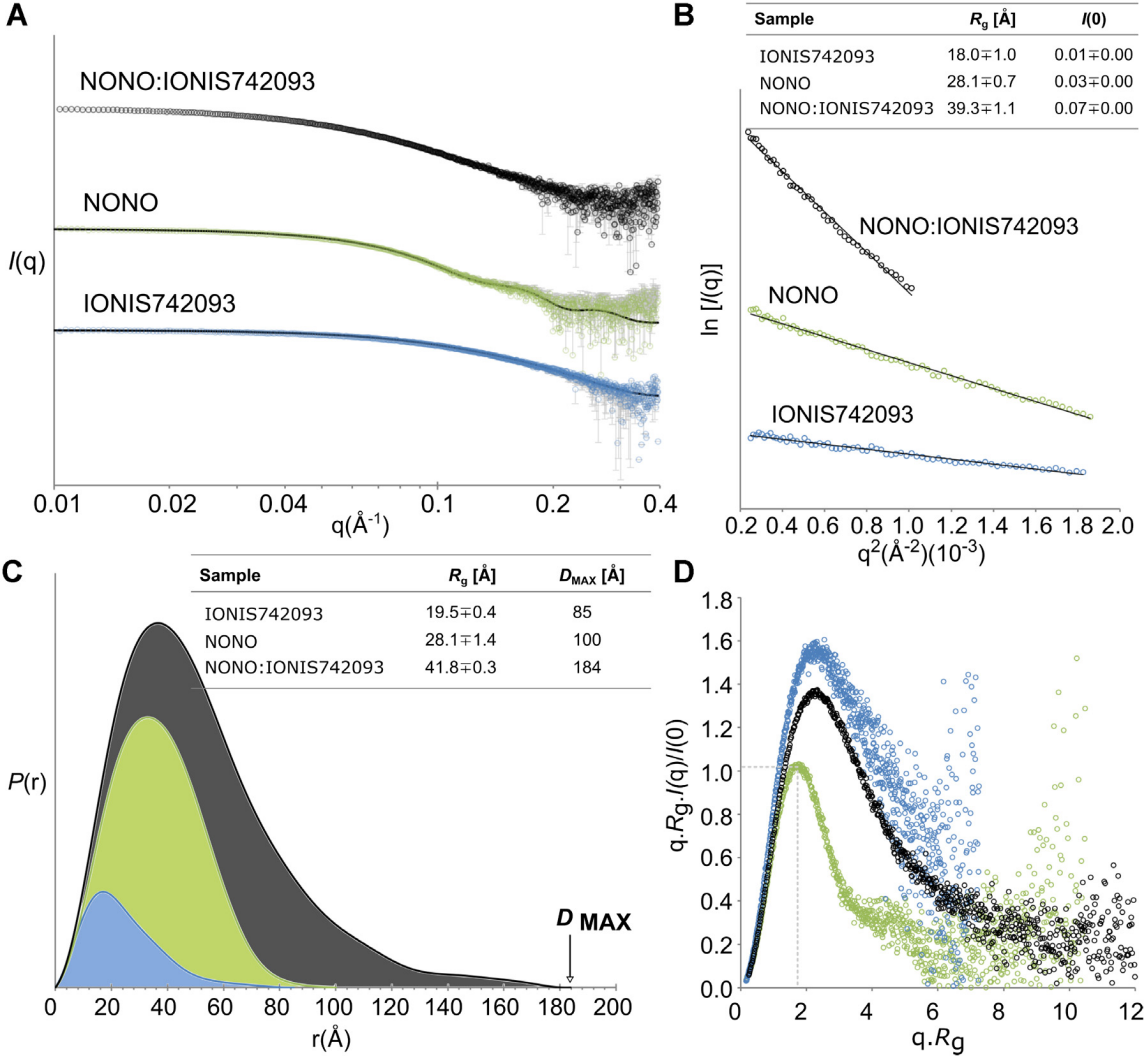
SAXS analysis of the NONO-DBHS homodimer, IONIS742093 and NONO:IONIS742093 complex. NONO data as published in (38). (**A**) Raw *I*(*q*) scattering data for NONO (green), IONIS742093 (blue) and NONO:IONIS742093 complex (black) plotted against the scattering angle *q* given in units of inverse Å (error bars, mean ± SD). The predicted scattering curves derived from models of NONO homodimer (5IFM) and bacterial group II intron (2M57) are overlaid with the NONO and IONIS742093 scattering data, respectively (solid black lines). (**B**) Guinier plot showing the reciprocal space derived radius of gyration (*R*_g_) given in units of Å and the forward scattering vector, *I*(0). (**C**) Pairwise distribution [*P*(*r*)] profiles for the three samples plotted against *r* in units of Å. The real space derived *R*_g_ and *D*_MAX_ are shown in a table. (**D**) Normalized Kratky plots for the three samples, where the intersection of *qR*_g_^2^ and *qR*_g_ is denoted by a dashed grey line.

To further understand the mechanism of NONO homodimer binding to IONIS742093, we prepared NONO, IONIS742093 and the NONO:IONIS742093 complex for SEC-SY-SAXS (48). The raw *I*(*q*) versus *q* scattering data are shown with theoretical scattering curves fitted to the data where appropriate (Figure 4A). The Guinier region and calculated radius of gyration (*R*_g_) for each data set are shown alongside the distance distribution [*P*(*r*)], function, maximum dimension (*D*_max_) and normalized (dimensionless) Kratky plot (Figure 4B–D). The structural parameters for each sample are summarized in Supplementary Table S1.

### NONO homodimers bind ASOs in an elongated complex

The solution scattering of NONO is consistent with a single crystallographically determined homodimer (38) (Figure 4A, *χ*-value = 1.04). IONIS742093 is a 20-nucleotide 5-10-5 ‘gapmer’ PS-ASO (Supplementary Figure S4). Solution scattering of IONIS742093 revealed a reciprocal space *R*_g_ of 18.0 Å, a real space *R*_g_ of 19.5 Å, a unimodal *P*(*r*) function and associated *D*_max_ of 85 Å (Figure 4B and C). These parameters, along with a non-parabolic curve in the normalized Kratky plot, hyperbolic rise in the Porod–Debye plot and plateau in the Kratky–Debye plot (Supplementary Figure S5), are indicative of a highly flexible prolate particle (63). Using the power-law relationships between *Q*_R_ and particle mass for RNA (56) revealed that IONIS742093 was ∼14.4 kDa, double the expected monomeric mass of 7.1 kDa (Supplementary Table S1). Searching the Protein Data Bank for structural neighbours identified that IONIS742093 solution scattering was most consistent with the 35-nt stem-loop domain 5 of the bacterial group II intron from *Azotobacter vinelandii* (*χ*-value = 0.94; PDB code: 2M57) (Figure 4A and Supplementary Figure S4B). Taken together, the SAXS data for IONIS742093 suggests that the ASO adopts a dimeric structure to produce a prolate particle that is consistent with a 35-nt stem-loop, albeit slightly longer.

Complexing NONO with IONIS742093, eluting over a gel filtration column (Supplementary Figure S6) while collecting SAXS data (Figure 4A), describes a particle with a reciprocal space/real space *R*_g_ of 39.3/41.8 Å, a significant increase compared to apo-NONO (*R*_g_ = 28.1 Å) (Supplementary Table S1). Consistent with this, the unimodal *P*(*r*) function for NONO:IONIS742093 describes a particle with a *D*_max_ of 184 Å (Figure 4C), which together with the *R*_g_ suggested an overall globular structure with elongated segments. In contrast to apo-NONO, the normalized Kratky plot for NONO:IONIS742093 reached a maximum at 2.2 *qR*_g_ gradually converging to zero at higher *qR*_g_ (Figure 4D), indicative of some degree of flexibility within a relatively compact particle. Further flexibility analysis by use of the Porod–Debye plot illustrated an asymptotic trend to a plateau that is consistent with a compact particle (Supplementary Figure S5). However, complexing NONO with IONIS742093 decreased the Porod exponent (*P*_X_) from 4.3 to 3.6 (Supplementary Table S1), which together with the flexibility analysis suggested that the complex possessed some elements of disorder. Determining an approximate molecular mass of the complex using the power-law relationship for protein (56) gave a mass range of 84.0–86.4 kDa, suggestive of four IONIS742093 molecules per NONO homodimer (Supplementary Table S1). Taken together, the SAXS reciprocal and real space structural parameters describe the NONO:IONIS742093 complex as four copies of IONIS742093 associated with a NONO homodimer in an elongated biphasic particle.

### Modelling the NONO:IONIS742093 complex

**Figure 5.**
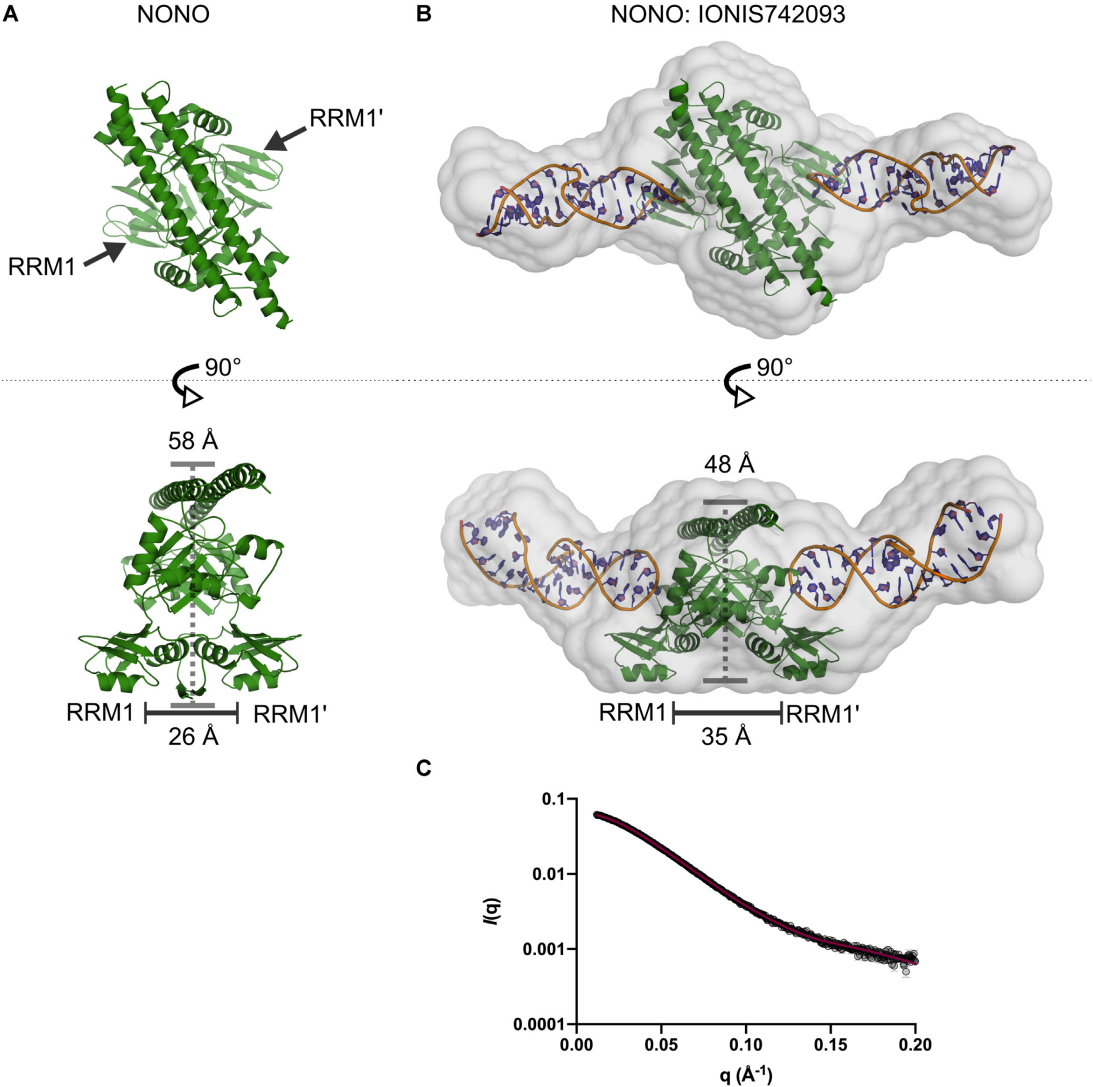
Solution structure of the NONO:IONIS742093 complex derived from SAXS data. (**A**) The X-ray crystal structure of a single NONO homodimer illustrated over two orthogonal perspectives highlighting the positions of RRM1 and RRM1′. (**B**) *Ab initio* reconstructions of the NONO:IONIS742093 complex calculated from SAXS data shown as a grey molecular envelope over two orthogonal perspectives. Superposed is a rigid body model for the NONO:IONIS742093 complex derived from SAXS data. The NONO homodimer is coloured green and the model for IONIS742093 (2M57) coloured blue and orange. The distances between the CCs, β-clasp and RRMs are shown to highlight the dimer compression upon binding. (**C**) Fit of refined model (red) to data (circles).

To better understand the solution state of the NONO:IONIS742093 complex, we modelled the complex assuming two copies of the 35-nt stem-loop domain 5 of the bacterial group II intron (PDB code: 2M57) appropriately described IONIS742093. A single crystallographic NONO homodimer (Figure 5A) defined as a rigid body with two copies of 2M57 converged on a solution that fit the data poorly (*χ* = 2.41). Given the observed cooperativity and requirement for RRM1, we reasoned that RRM1 might undergo some rigid body motion to accommodate the nucleic acid. To model this, a restraint of 5.0–7.5 Å was used to describe the flexible linker between RRM1 and RRM2 (Figure 5B). A good rigid body fit to the NONO:IONIS742093 data was obtained that described an overall compression of the dimer where RRM1 and RRM1′ shift laterally and upwards from under the core of the dimer (*χ* = 1.69) (Figure 5B). Consistent but independent of this observation, *ab initio* reconstruction produced a molecular envelope characterized by a globular core with elongated segments (*χ* = 1.49) (Figure 5B). Taken together, these data are in good agreement with a solution structure that describes a reorientation of the RRM1 domains to accommodate the duplexed IONIS742093 associating above the β-sheet surface of RRM1.

## DISCUSSION

Human DBHS paralogues are involved in almost every step of gene regulation, often sharing overlapping functions. However, emerging evidence suggests that the individual DBHS paralogues have non-redundant roles in both their transcriptional and post-transcriptional activity (12,64). Thus, in spite of their remarkable similarity, the functional context of a DBHS dimer may, in part, be dictated by the dimerization state. Consistent with this, the relative abundance of SFPQ, NONO or PSPC1 varies with cell type and this in turn influences dimer partitioning (65,66). Our study describes the X-ray crystal structures of homodimeric NONO and PSPC1 and provides novel insights into the nature of combinatorial DBHS protein dimerization and RNA binding.

The structures of homodimeric PSPC1 and NONO are broadly similar to their heterodimeric counterpart. However, key differences are the relative conformations of the RRM1 and CC, and the presence of an N-terminal β-clasp. The presence of the well-ordered β-clasp is strikingly different from the disorder seen in the N-terminal residues of the PSPC1/NONO heterodimer (16) and the short α-helix within the SFPQ homodimer (18). As a result of the N-terminal antiparallel β-clasp and the C-terminal antiparallel arrangement of the CC domains, the PSPC1 and NONO homodimers are intertwined to an extent that there is a full 360° left-handed twist of one chain around the other. With such an extensive interaction interface, it is quite remarkable that DBHS proteins are able to readily exchange dimerization partner in a cellular context. One clue as to the variable regions involved in partner swapping lies in the suite of dimers within the NONO crystal structure (Figure 1). These alternative NONO conformations reveal a structural dynamic in the positioning of the NOPS domain and the distal CC′. In NONO, the subtle conformational variability within the core dimerization interface was correlated with the two alternative conformations (W-1 and W-2) adopted by W271. It was previously noted that the structural plasticity of this region may drive the observed preference for heterodimerization in DBHS proteins (14,16). Examining the dimerization interface of each DBHS dimer state reveals that PSPC1/NONO possesses an asymmetric complementarity in its electrostatic and hydrophobic interactions within the dimerization interface, contacts that are absent in the homodimeric structures (Figure 2). Furthermore, the sequences of NONO and SFPQ, coupled with the homodimeric crystal structures, would suggest that a heterodimer formed between NONO and SFPQ would have a more energetically favourable interface than the individual homodimers. Collectively, these structural observations lead us to hypothesize that the core set of contacts in the dimerization interface, coupled with varying flexibility, engenders a preference for heterodimerization in DBHS proteins. While this is consistent with observations *in vitro* and *in vivo*, the *K*_D_ values for only some interacting DBHS protein pairs are known [the SFPQ homo- and heterodimers (14)]. However, we note that the extended CC and low-complexity domains may well influence the propensity to form homo- or heterodimers. Furthermore, post-translation modifications, interaction partner and relative expression levels could all influence dimerization.

The structural variability of the dimerization interface in the six independent copies of the NONO homodimer in the crystal structure may indicate how structural plasticity can drive DBHS dimers to readily exchange partner. Interestingly, the broad flexibility of the NONO homodimer appears to be destabilizing given that in the absence of l-proline, NONO rapidly aggregates and precipitates (38). The crystal structure of the NONO homodimer illustrates that l-proline is involved in a site-specific interaction with F218 of RRM2 (Supplementary Figure S2), where it potentially limits the local conformational variability and stabilizes the protein. Alternatively, l-proline may be acting in a more generalized capacity as a cosmotrope where it modulates the ordered shell of water molecules solubilizing a protein (67,68). However, a closer examination of the l-proline binding pocket reveals the proximity of two highly conserved glutamate residues (E297 and E301) within the distal CC′ that do not contribute to the dimerization interface. It is tempting to suggest that the ‘groove’ occupied by l-proline may serve as an interaction site for nucleic acid or other cofactors that influence dimerization.

### Novel insights into DBHS protein nucleic acid binding

Consistent with previous observations (23,25,37,69,70), we demonstrated that both PSPC1 and NONO have a broad specificity for single-stranded RNA but exhibit the greatest affinity for G- and U-rich sequences. Notably, we demonstrate that the interaction with simple homo-ribopolymers requires the canonical RRM1 and, when present, binding occurs in a cooperative manner, suggesting that a NONO homodimer accommodates two or more RNAs at dependent binding sites. The binding events are relatively weak when compared to other RNA binding proteins that use a canonical RRM (71). The low affinity could be explained by the absence of the CC oligomerization domain that, when present, may increase the avidity for unstructured nucleic acids (18). However, we do observe that NONO binds with strong affinity to 2′-F-PS-ASOs, consistent with previous observations (32,33). Additionally, we have shown that this interaction, like the homo-ribopolymers, is dependent on the presence of RRM1. Furthermore, when given the same nucleotide sequence with 2′-cEt modifications, the binding affinity was markedly lower, suggesting that the high affinity is provided by the 2′-F modification.

Our *in vitro* measurements show more extreme discrimination between 2′-F and 2′-cEt PS-ASOs, and also between intact and ΔRRM1 variants of NONO, than those observed from HeLa cell extracts using a quantitative BRET assay (36). A likely cause for the lower discrimination in cell extracts is the presence of wild-type DBHS proteins, which can all dimerize and oligomerize with overexpressed proteins *in vivo*.

Our data indicate that the 2′-F-PS-ASO exists as a structured duplex in solution. Secondary structure predictions using the nucleotide sequence indicated that some secondary structure may exist; however, it is well known that 2′-F modifications have a stabilizing effect on base stacking and Watson–Crick base pairing (72,73). Thus, it is possible that the binding of the NONO homodimer to the 2′-F-ASO is in fact dependent on its structure, stabilized by the presence of 2′-F modifications, rather than any inherent affinity for 2′-F modifications. Interestingly, our binding studies indicate that RRM1 is required for the interaction with the structured oligonucleotide. As described earlier, the first RRM in DBHS proteins strongly resembles a canonical single-stranded RNA binding domain (16,17,71). Thus, it is likely that a proximal domain (e.g. NOPS and RRM2) contributes to the recognition of secondary structure.

SEC-SY-SAXS-derived models of the NONO homodimer in complex with 2′-F-ASO describe a NONO homodimer forming a 1:2 complex with duplexed 2′-F-ASO. The solution structures reveal the duplexed oligonucleotide associating above the highly conserved β-sheet surface of RRM1, consistent with the binding data. The association above the β-sheet surface of RRM1 requires that RRM1 and RRM1′ shift laterally from under the core of the dimer. It should be noted that the *χ*-values deviate from 1.0, which may be attributed to the use of rigid bodies in modelling and that modelling was carried out against low-resolution solution data. Nevertheless, the fit to the data was improved by allowing for the subtle domain re-arrangement of the N-terminal RRM1. The NONO and PSPC1 homodimers possess a novel N-terminal β-clasp that, to facilitate the RRM1 movement, must presumably ‘unlock’ for RRM1 to shift out from under the core to allow the re-arrangement necessary to accommodate the ASO. The implication that an N-terminal DBHS region might regulate nucleic acid binding ability has been noted for SFPQ (26,74). The N-terminal DBD of SFPQ associates with double-stranded DNA. However, upon RNA binding to potentially RRM1 or RRM2, SFPQ is released from double-stranded DNA, perhaps by a similar allosteric effect that translates to the DBD (18,26,74). Furthermore, post-translational modifications in the N-terminus of NONO do influence RNA binding (23), perhaps by fixing the N-terminal RRMs in a locked state. Interestingly, the movement of the N-terminal RRMs out from beneath the dimer core brings them closer to the highly conserved β2–β3 loop of RRM2. Interestingly, quasi-RRMs, those that lack any conserved aromatic residues on the β-sheet surface, can employ the β1–α1, β2–β3 and/or α2–β4 loops for RNA interaction (71). While it is appealing to suggest that the highly conserved DBHS β2–β3 loops may interact with grooves within the structured RNA, similar to RBMY (75), further investigation is required to define the role of this loop. These observations are in agreement with Vickers and Crooke (36) who demonstrated that both NONOΔRRM1 and NONOΔRRM2 variants demonstrated reduced ASO binding *in vivo*, whereas RRM1 showed much tighter binding than RRM2 to 2′-F-PS-ASO (76), although we note that the deletion of RRM2 has such a catastrophic effect on the essential dimerization of a DBHS protein that it cannot be considered a functional protein.

The reported interaction between NONO and 2′-F-PS-ASOs leads to a reduction in the nuclear abundance of NONO, likely due to targeted protein degradation (33). While the binding of the 2′-F-PS-ASOs to the NONO homodimer described in this study did not appear to compromise the integrity of the dimer, it is possible that the large-scale nuclear mRNP aggregates formed with ASO treatment elicit a defensive response to mitigate the formation of pathological aggregates, or that PS-ASO binding may affect the NONO interaction with other partner proteins (77). Ultimately, further investigation is required to explore what effect 2′-F-ASO binding to NONO containing dimers has within a cellular context.

The X-ray crystal structures described herein provide further evidence for conformational variability within DBHS protein dimer states. Specifically, we have now observed the N-terminal RRM, NOPS domain and distal CC in different conformations. While the consequences of the inferred domain and side chain motions are unclear, cumulative evidence would suggest that the motion is linked to dimerization propensity. This work also presents the first structural study that details the roles of RRM1 and RRM2 in DBHS nucleic acid interaction. The NONO homodimers form a highly specific 1:2 complex with duplexed ASOs, undergoing a subtle conformational change that facilitates cooperativity and simultaneous recognition of nucleic acid above the canonical RRM1. These data make significant contributions to our understanding of nucleic acid interactions and coupled with the X-ray crystal structures of the PSPC1 and NONO homodimers pave the way to explore the interactions further.

## DATA AVAILABILITY

The atomic coordinates and structure factors for the NONO homodimer and PSPC1 homodimer have been deposited in the Protein Data Bank under the accession codes 5IFM and 5IFN, respectively. SAXS data and fits were deposited at SASBDB under the accession codes SASDMR6, SASDMS6, SASDMT6 and project 1489.

## Supplementary Material

gkab1216_Supplemental_File

## Notes

Present address: Gavin J. Knott, Monash Biomedicine Discovery Institute, Department of Biochemistry and Molecular Biology, Monash University, Victoria 3800, Australia.

Present address: Daniel M. Passon, Targenomix GmbH, Am Mühlenberg 11, 14476 Potsdam, Germany.
