# Supplementary material for: Structural basis of dimerization and nucleic acid binding of human DBHS proteins NONO and PSPC1
Source: Nucleic Acids Res. 2021 Dec 14;50(1):522–35. doi: 10.1093/nar/gkab1216 (PMC8754649; doi:10.1093/nar/gkab1216)
Supplement: gkab1216_Supplemental_File [file gkab1216_supplemental_file.pdf]

SUPPLEMENTARY INFORMATION

Structural Basis of Dimerisation and Nucleic Acid Binding of Human DBHS Proteins NONO and PSPC1

Gavin J. Knott[1,2], Yee Seng Chong[1], Daniel M. Passon[1,3], Xue-hai Liang[4], Evelyne Deplazes[5], Maria R. Conte[6], Andrew C. Marshall[1], Mihwa Lee[7], Archa H. Fox[1,8], and Charles S. Bond[1*]

[1] School of Molecular Sciences, The University of Western Australia, Crawley, Western Australia, 6009, Australia.

[2] Current address: Biomedicine Discovery Institute, Monash University, 19 Innovation Walk, Clayton, Victoria, 3800, Australia

[3] Current address: Targenomix GmbH, Am Mühlenberg 11, 14476 Potsdam, Germany

[4] Department of Core Antisense Research, IONIS Pharmaceuticals Inc. 2855 Gazelle Court, Carlsbad, CA 92010, USA.

[5] School of Chemistry and Molecular Biosciences, The University of Queensland, St Lucia, 4072, Queensland, Australia

[6] Randall Division of Cell and Molecular Biophysics, King's College London, New Hunt's House, Guy's Campus, London SE1 1UL, UK,

[7] Department of Biochemistry and Genetics, La Trobe Institute for Molecular Science, La Trobe University, Bundoora, Victoria, 3086, Australia

[8] School of Human Sciences, The University of Western Australia, Crawley, Western Australia, 6009, Australia,

[*] Correspondence should be addressed to C.S.B (charles.bond@uwa.edu.au)

Supplementary Table 1 – SAXS data collection and analysis statistics

	NONO	ASO	NONO: IONIS742093
**Data collection**			
q range ($Å^{-1}$)	0.006 – 0.375	0.006 – 0.375	0.006 – 0.375
**Structural parameters**			
$I(0)$ (cm^{-1}) [$P(r)$]	0.03 ± 0.00	0.01 ± 0.00	0.07 ± 0.00
R_g (Å) [P(r)]	28.1 ± 1.4	19.5 ± 0.4	41.8 ± 0.3
$I(0)$ (cm^{-1}) [Guinier]	0.03 ± 0.00	0.01 ± 0.00	0.07 ± 0.00
R_g (Å) [Guinier]	28.1 ± 0.7	18.0 ± 1.0	39.3 ± 1.1
D_{max} (Å)	100	85	184
**Molecular-mass**			
Porod exponent (P_X)	4.3	1.9	3.6
Porod invariant (V_p)($Å^3$)	94,253	15,230	152,800
Volume-of-correlation (V_c)	461.66	160.0	666.66
Theoretical-mass (kDa)	30.4	7.1	-
Calculated-mass (kDa)*	61.6	14.4	86.4†
Stoichiometry (n)	Dimer	Dimer	~ 4 RNA / Dimer

a

* Masses are calculated using power-law relationship with V_c for protein or RNA only scattering for NONO and ASO respectively.

† Masses are calculated using power-law relationship with V_c for protein scattering.

Supplementary Table 2 – Clustering NONO chains by variation within the NOPS domain.

NONO Chain	Partner Chain	R.M.S.D[a] (Å)	$\chi 1$ (°)†	$\chi 2$ (°)†	B-factor (Å^3)‡	Cluster ID
A	B	0.64	- 177.0	- 103.0	58.3	W-1
B*	A	0.00	- 87.0	107.0	46.9	W-2
C	D	0.73	- 85.0	101.0	64.4	W-2
D*	C	0.36	- 83.0	113.0	46.1	W-2
E	F	0.70	- 178.0	- 103.0	83.3	W-1
F	E	0.78	- 177.0	- 104.0	91.6	W-1
G	H	0.97	- 178.0	- 103.0	73.0	W-1
H	G	0.72	- 177.0	- 103.0	70.2	W-1
I	J	0.75	- 176.0	- 102.0	77.1	W-1
J	I	0.33	- 83.0	103.0	64.8	W-2
K	L	0.45	- 87.0	105.0	60.6	W-2
L	K	0.39	- 87.0	104.0	74.9	W-2

‡ Average B-factor of W271 calculated with *BAVERAGE*.

R.M.S.D value for the NOPS domain (aa 227-275) of each chain relative to Chain-B.

* Chains that have a L-proline bound within the dimerisation interface.

† Torsion angles $\chi 1$ and $\chi 2$ for W271.

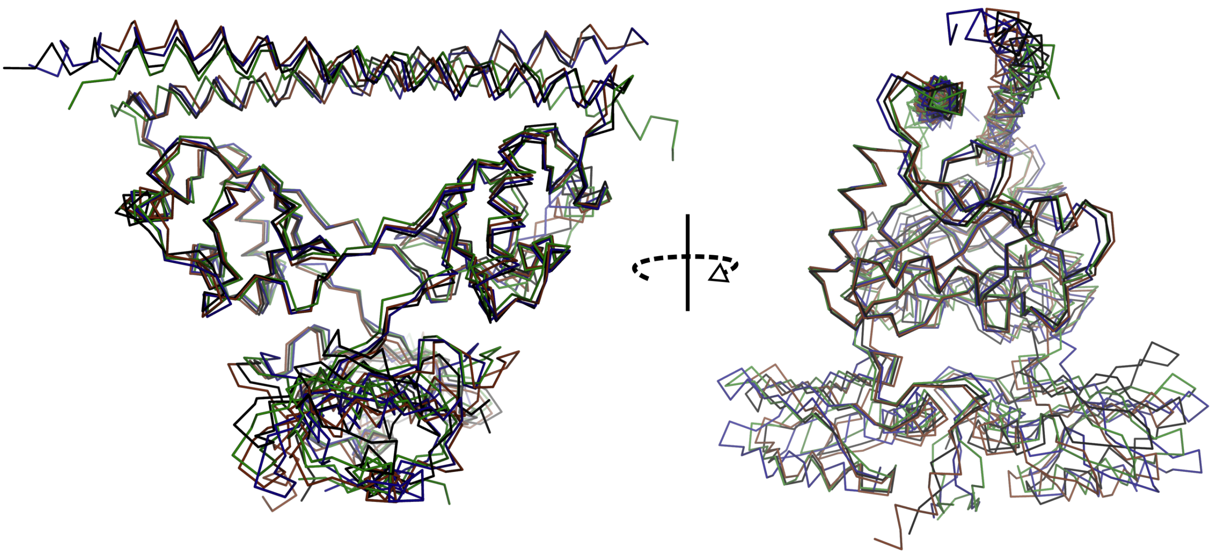

Supplementary Figure 1 - DBHS Dimer Structure Comparison. Ribbon superposition of NONO (53-312) homodimer (chains AB, green) with the PSPC1 (60-320) homodimer (blue), PSPC1/NONO (60-320/53-312) heterodimer (black), and SFPQ (276- 535) homodimer (maroon).

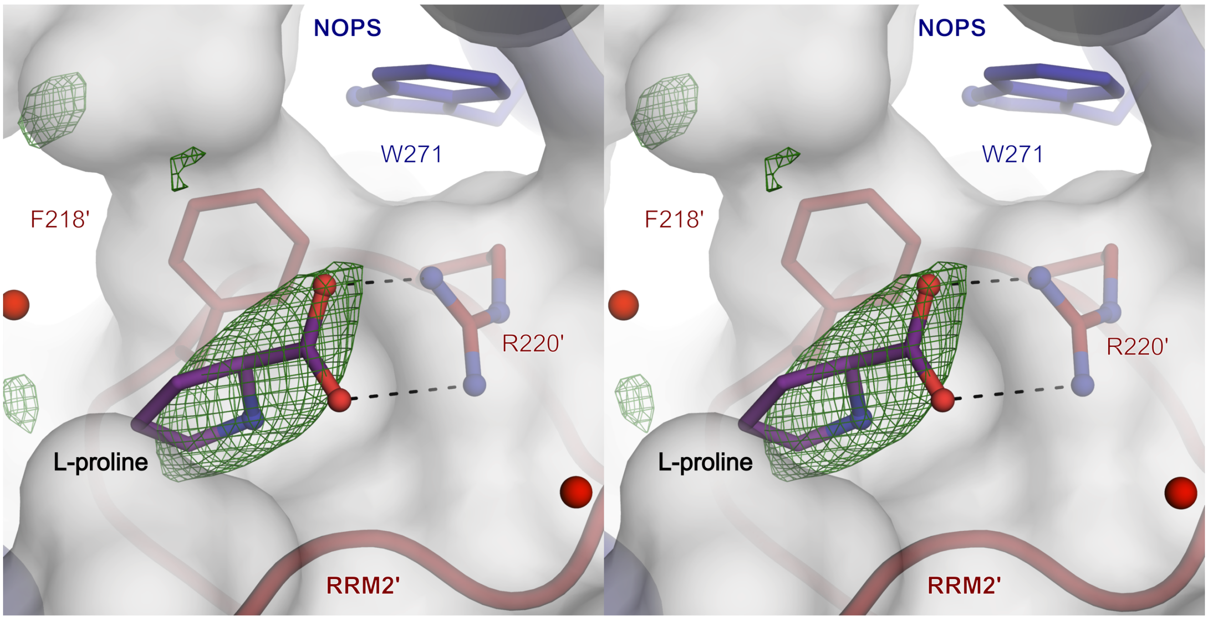

Supplementary Figure 2 – Stereo view of the L-proline binding pocket within the NONO homodimer. The L-proline (purple) is illustrated interacting with residues within the partnered RRM2 (RRM2', red cartoon) in a solvent accessible pocket (surface). The positive omit F_{obs}-F_{calc} isomesh (green) after omitting L-proline from refinement is shown contoured to 3.0 σ over a 10.0-Å spherical radius relative to the L-proline.

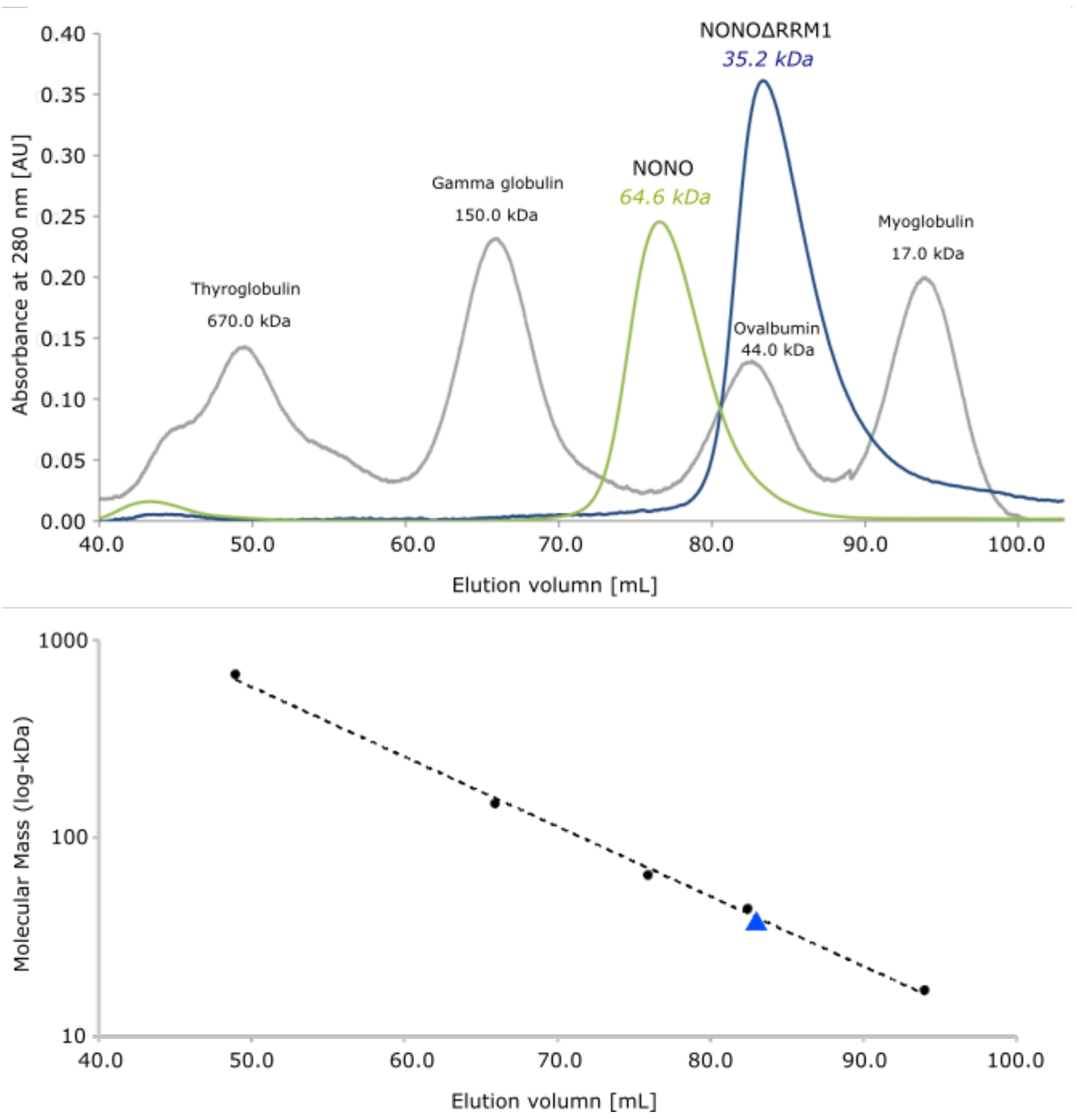

Supplementary Figure 3 - Size exclusion chromatography trace indicating NONOΔRRM1 is globular and dimeric. The size exclusion profiles of NONOΔRRM1 (blue) are overlayed with the NONO homodimer (green) and gel filtration standards (black). The elution volume (mL) is plotted against absorbance at 280 nm (in arbitrary units, AU). The known molecular weights (kDa) for the standards are indicated above the corresponding peak. Molecular weights for NONO and NONOΔRRM1 are indicated. Standard curve constructed using all data except NONOΔRRM1 which is plotted as a blue triangle at the predicted molecular mass of a dimer.

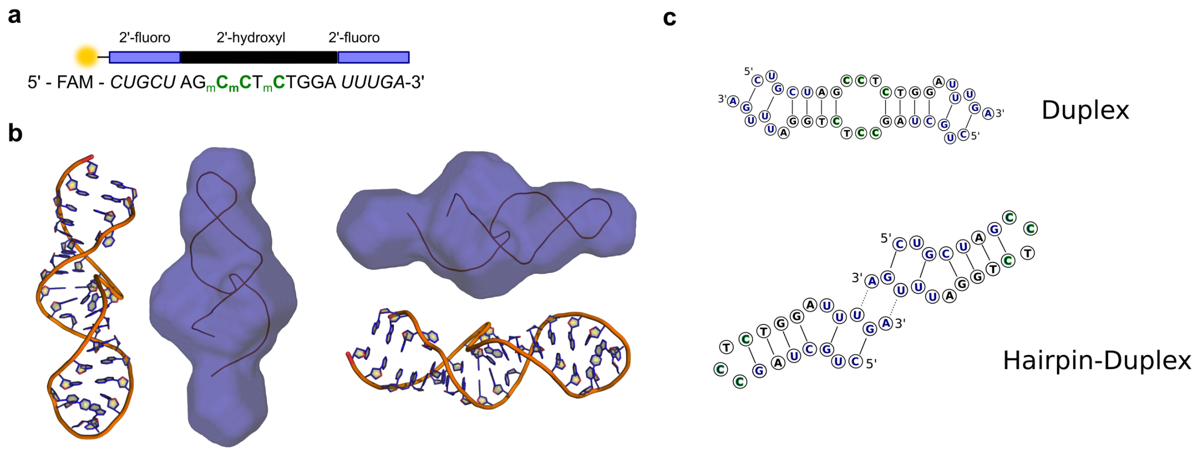

Supplementary Figure 4 – SAXS analysis of IONIS742093. (a) Primary sequence of IONIS742093 showing the 5-10-5 gapmer design. The primary sequence is shown below a schematic of the ASO where 2′-fluoro nucleotides (blue) are flanking the 2′-OH nucleotides (black). Methylated cytosine residues are shown in green. (b) *Ab initio* reconstruction of IONIS742093 shown over two orthogonal views with the NMR structure of 2M57 fitted within the molecular envelope. (c) Secondary structure predictions for IONIS742093 as a duplex or hairpin-duplex. Nucleotides are coloured as in (a).

A

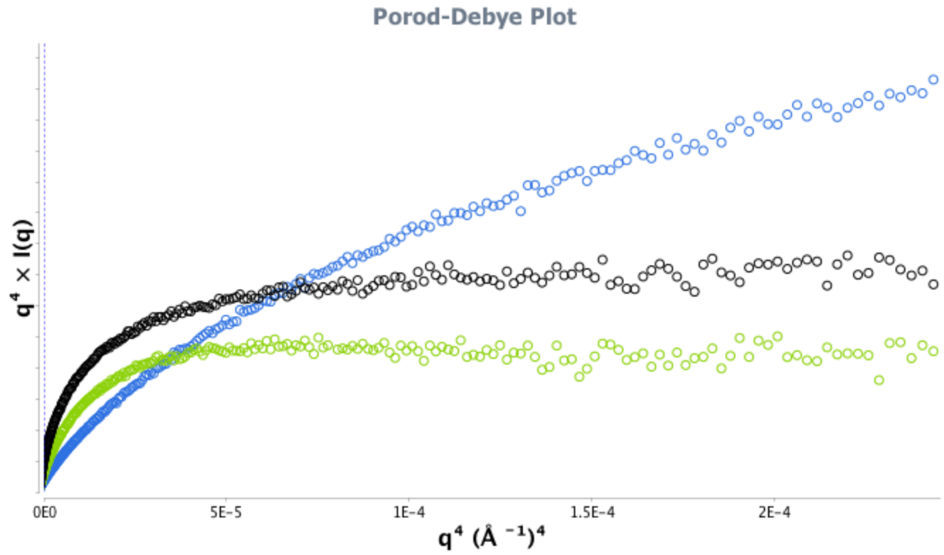

B

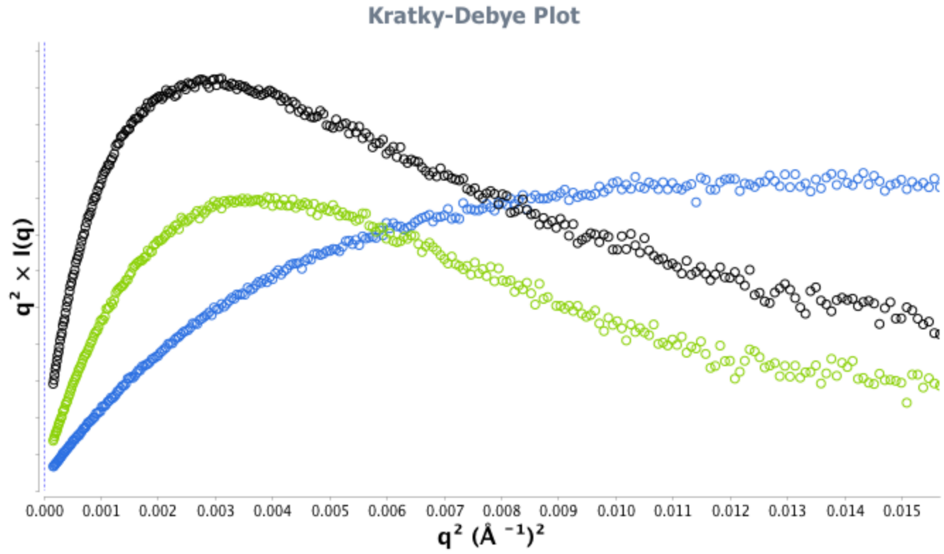

Supplementary Figure 5 - SCÅTTER flexibility analysis of NONO. Apo-NONO homodimer (green), apo- 2'-F-antisense oligonucleotide (ASO) (IONIS742093) (blue) and NONO: ASO complex (black and purple for NONO: ASO α/β respectively). (a) Porod-Debye plot and (b) Kratky-Debye plots are shown to a q of 0.122 $Å^{-1}$.

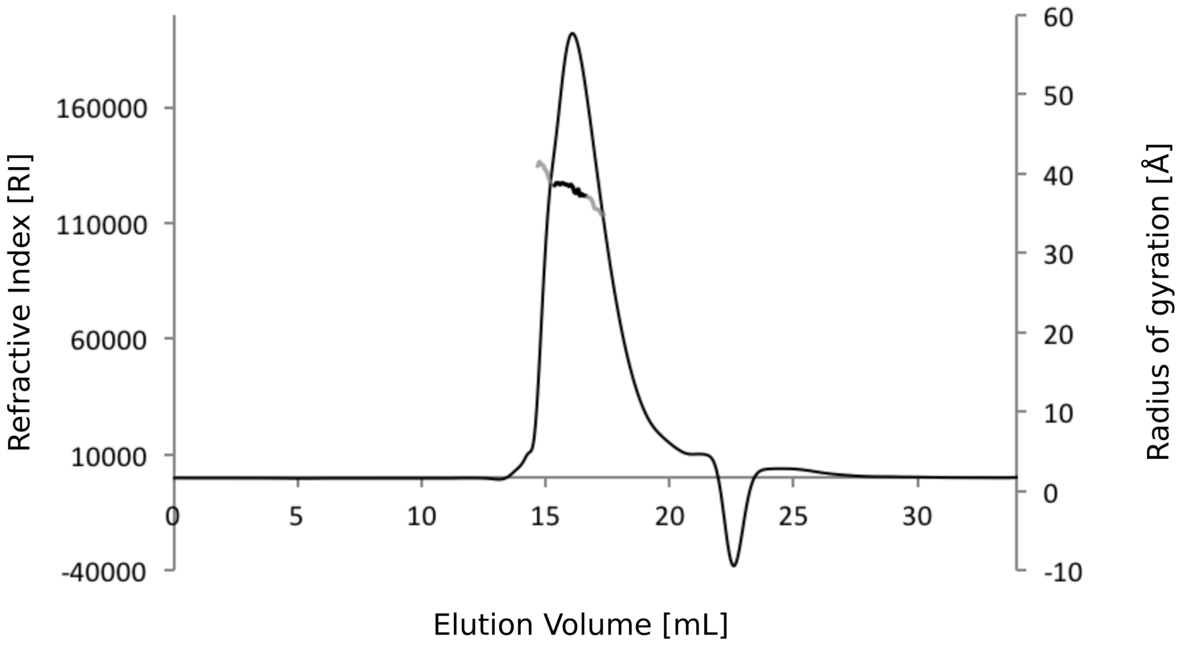

Supplementary Figure 6 - Size exclusion chromatography trace for NONO in complex with the 2'-F-PS-ASO (IONIS742093). Collected during SEC-SY-SAXS at the SAXS/WAXS beamline of the Australian Synchrotron. The SAXS-derived reciprocal space radius of gyration (R_g) is overlayed with the refractive index.
